# Follow-up Psychiatric Care and Risk of Readmission in Patients with Serious Mental Illness in State Funded or Operated Facilities

**DOI:** 10.1007/s11126-021-09957-0

**Published:** 2021-10-25

**Authors:** Linda Hermer, Thomas Nephew, Kenona Southwell

**Affiliations:** Eagle Technologies, Inc, Arlington, VA USA

**Keywords:** Schizophrenia, Bipolar disorder, Major depressive disorder, Hospitalization, Outpatient care, Readmissions

## Abstract

Receipt of outpatient treatment within 30 days of discharge from psychiatric hospitalization is an established quality indicator; however, there is scant, mixed evidence as to whether it reduces the risk of readmission. We evaluated this question in patients hospitalized for schizophrenic, bipolar or depressive disorders using the Mental Health Treatment Episode Data Set (MH-TEDS), comprising patients in state-funded or -operated facilities and programs. We performed a 6-month, retrospective longitudinal cohort study including 44,761 patients with schizophrenic disorders, 45,413 patients with bipolar disorders, and 74,995 patients with depressive disorders with an index hospitalization between 2014 and 2018, stratified by whether they had at least one outpatient treatment admission in the first 30 days post-discharge. We used multivariable logistic regression to assess risk of readmission during days 31–180. We found that less than 10 percent of patients in the three cohorts received the recommended follow-up outpatient care. Furthermore, we found that schizophrenic and bipolar patients who did receive such care were no less likely to be readmitted than those not receiving such care (AOR = 0.96, 95% CI 0.87–1.06; AOR 1.06, 955 CI 0.98–1.14), and patients with depressive disorders receiving such care were more likely to be readmitted (AOR = 1.14, 95% CI 1.07–1.22). Thus, few patients received follow-up outpatient care within 30 days of discharge. When it occurred, such outpatient care was either not linked to reduced readmissions or was associated with increased readmissions. These findings suggest the need for more effective care processes in state-funded or -operated facilities.

## Introduction

Adverse outcomes following psychiatric hospitalization are relatively common. For example, in a recent meta-analysis of 100 studies of post-discharge suicide conducted in countries from five continents, the suicide rate was approximately 100 times the global average in the first three months post-discharge. The suicide rate was even higher—200 times the global average—for patients admitted with suicidal ideation [1]. Rapid readmissions are also frequent. A recent study found a 20.9 percent all-cause readmission rate over the first 30 days following discharge from psychiatric hospitalization [2].

The Healthcare Effectiveness Data and Information Set (HEDIS) is a widely used set of performance measures in the healthcare industry and by government agencies, developed by the National Committee for Quality Assurance (NCQA). An established HEDIS quality indicator recommends that psychiatric inpatients receive follow-up outpatient care within 30 days of discharge [3]. The rationale for this measure is that there are more than two million psychiatric hospitalizations each year in the United States and that psychiatric inpatients are vulnerable after discharge [3]. There are many potential benefits of timely outpatient care following discharge for psychiatric hospitalization, such as tighter linkage between inpatient and outpatient care plans and continuity of pharmacotherapy [4].

However, the few U.S.-based studies that have examined the effectiveness of such follow-up care at preventing readmissions have found relatively little evidence of a benefit. Marcus et al. [5] studied schizophrenic and bipolar patient cohorts, finding a modest reduction in readmissions through 120 days post-discharge. Ilgen et al. [6] examined veterans with co-occurring mental health and substance use disorders and found no effect of follow-up mental health care on 90-day readmissions; however, they did find a reduction in readmissions following outpatient substance use care. Pfeiffer et al. [7] studied veterans who had been hospitalized for depression and found no reduction in 90-day readmissions among patients receiving the recommended follow-up care. In apparent contrast, the non-U.S.-based studies that have been performed have provided more compelling evidence that timely outpatient visits reduce readmissions. For instance, in a recent study of Japanese bipolar and schizophrenic patients who had a high rate of follow-up outpatient care, Okumura et al. [8] found that the few patients who did not receive the recommended follow-up care after psychiatric hospitalization were substantially more likely to be readmitted. Similarly, in a study of Taiwanese schizophrenic patients, Lin and Lee [9] found both a high rate of post-hospitalization follow-up visits and reduced readmissions in those receiving prompt follow-up outpatient care.

### Purpose of the Study

We examined whether outpatient treatment within 30 days of discharge was associated with reduced readmissions in patients with serious mental illness (SMI, [10])—specifically, those patients with schizophrenic, bipolar or depressive disorders—with or without co-occurring substance use disorder. A distinguishing feature of this study was that we used a large data set of patients in state-funded or -operated treatment programs. We conducted a retrospective longitudinal cohort analysis in which we examined readmission risk during the 6 months post-discharge. Some previous investigations of this question followed patients for either three months [6, 7] or four months [5] post-discharge. However, one prior study finding a benefit employed a 6-month follow-up period [8] and three prior studies showed at least some evidence of a benefit as long as one year post-discharge [6, 9, 11]. Thus, we employed a 6-month window for our analysis of the effects of follow-up care.

## Methods

### Data Set Construction and Sample Selection

The study used 2014–2019 MH-TEDS data. MH-TEDS comprises data on individuals receiving mental health treatment or services that are funded or operated by state mental health agencies. MH-TEDS is a rich data source on this important and large patient population that is underrepresented in the literature. Between 2014 and 2019, 13 states and other jurisdictions contributed data to MH-TEDS: Connecticut, the District of Columbia, Delaware, Florida, Louisiana, Michigan, Mississippi, North Carolina, Nebraska, Oklahoma, Pennsylvania, Puerto Rico, and West Virginia. Because states report only de-identified data, no informed consent or Institutional Review Board review was necessary.

For each year, MH-TEDS includes an admissions data set and a discharges data set. In MH-TEDS, an “admission” is intended to represent the beginning of treatment and a “discharge,” the ending of treatment [12]. For most states, admissions are the first day treatment was received. However, not all states may have administrative procedures to capture these data. In that case, they may use the pre-authorization start date for admissions [12]. Because there may be some variation across states in how these data are reported, we use the MH-TEDS terms “admissions” and “discharges” even for outpatient treatment.

We linked the admissions and discharges records and constructed a client-level, longitudinal data set of inpatient and outpatient treatment over the 6 months following discharge from patients’ index hospitalization. Residential treatment was rare and excluded from the analysis.

The sample included individuals in MH-TEDS who were 18 years or older, had an index hospitalization between 2014 and 2018, and were alive at least 6 months post-discharge. MH-TEDS does not include a primary diagnosis variable because not all states collect those data, but at least one discharge diagnosis must be listed and up to three can be provided. Thus, to focus on SMI, individuals were included if any of their index hospitalization discharge diagnoses were schizophrenic, bipolar, or depressive disorders. In cases where an individual had more than one eligible hospitalization, the first one was used.

Timely outpatient treatment could occur for any reason (e.g., medication management, psychotherapy, or substance use disorder treatment). The episode was required to have an admission date within 30 days of discharge from the index hospitalization. Based on their admission date, hospital readmissions were identified that occurred during the 6 months following discharge from the index hospitalization. Patients were excluded from the sample if they were readmitted within the first 30 days during which the recommended outpatient treatment was to occur.

### Independent Variables

The key predictor variable was whether patients had an admission for an outpatient treatment episode within 30 days of discharge from the index hospitalization. Patient characteristics included age group (18–24 years, 25–44 years, 45–64 years, and 65 years or older), sex, race (white, Black, and other/unknown), Hispanic ethnicity, number of mental health diagnoses, and co-occurring substance use disorder status. Characteristics of patients’ index hospitalization included length of stay (1–7 days, 8–30 days, and 31 or more days; the median length of stay was 7 days) and hospitalization facility type (state hospital versus another inpatient facility).

### Dependent Variables

The outcome studied was whether patients were readmitted within 31–180 days of discharge from the index hospitalization.

### Statistical Analysis

We analyzed each patient group (those with schizophrenic, bipolar, or depressive disorders) separately. First, we conducted a potential confounder analysis. We stratified each patient group by whether they had an admission for outpatient treatment within 30 days of discharge. Then, for frequencies, we conducted Wald chi-square tests to determine whether the two exposure groups differed. For continuous covariates (i.e. number of mental health diagnoses), we tested whether the variances were equal (in all cases they were) and then performed independent samples t-tests with pooled variance. Next, we computed the percentage of patients that had received an outpatient admission within 30 days of discharge and 6 months of discharge, and that had been readmitted within 6 months of discharge. Because patients who had been readmitted within 30 days of discharge were excluded, no readmissions in the first 30 days post-discharge were included. Finally, to test whether outpatient treatment within 30 days of discharge was associated with reduced readmissions, we performed logistic regression analyses that included the predictor variable and all covariates. The data met the assumptions for logistic regression in that there was independence and little multicollinearlity among the independent variables, there was a linear relationship between the independent variables and the log odds, and the sample size was large.

## Results

### Outpatient Mental Health Follow-up Admissions: Patient and Hospitalization Characteristics

Compared with schizophrenic disorder patients who did not receive a follow-up outpatient admission within 30 days of discharge (*N* = 40,448), those receiving such an admission (*N* = 4,313) were more likely to be younger, white, non-Hispanic or of unknown ethnicity, to have fewer diagnoses, and to be diagnosed with a co-occurring substance use disorder (Table 1). They were also more likely to have been hospitalized at a state hospital and to have had a longer inpatient length of stay.

**Table 1:** Sample demographics and hospitalization characteristics, by whether patients received the recommended post-discharge outpatient treatment. Percentages (or means and standard deviations where indicated) and *p*-values. Dxs: diagnoses

	Schizophrenia (*N* = 44,761)			Bipolar Disorder (*N* = 45,413)			Depression (*N* = 74,995)		
	No 30-Day Follow-Up Episode (*N* = 40,448)	30-Day Follow-Up Episode (*N* = 4,313)	*P*-Value	No 30-Day Follow-Up Episode (*N* = 41,418)	30-Day Follow-Up Episode (*N* = 3,995)	*P*-Value	No 30-Day Follow-Up Episode (*N* = 68,263)	30-Day Follow-Up Episode (*N* = 6,732)	*P*-Value
Age Group (Years)			< 0.0001			< 0.0001			< 0.0001
18–24	13.9	18.4		17.0	18.7		18.3	21.6	
25–44	47.4	48.5		54.0	54.8		49.0	49.4	
45–64	33.6	30.5		27.1	25.5		30.6	27.8	
65 +	5.1	2.6		1.9	0.9		2.2	1.2	
Sex			0.8			0.099			< 0.0001
Female	37.4	37.2		50.9	52.3		48.5	52.4	
Male	62.6	62.8		49.1	47.7		51.5	47.6	
Race			< 0.0001			0.0017			< 0.0001
Other/Unknown	10.9	11.3		9.0	9.4		10.1	10.8	
White	47.5	54.5		71.6	73.4		69.5	70.9	
Black	41.6	34.3		19.4	17.1		20.4	18.2	
Ethnicity			0.03			0.24			0.0012
Not Hispanic/Unknown	92.5	93.4		93.5	93.0		92.4	91.3	
Hispanic	7.5	6.6		6.6	7.0		7.6	8.7	
Number of Dxs			< 0.0001			< 0.0001			< 0.0001
(Mean (SD))	1.41 (0.67)	1.36 (0.62)		1.59 (0.75)	1.47 (0.68)		1.51 (0.72)	1.37 (0.61)	
Co-occurring SUD			< 0.0001			< 0.0001			< 0.0001
No	79.9	71.2		73.3	65.7		74.5	70.6	
Yes	20.1	28.8		26.7	34.3		25.5	29.4	
Inpatient Facility			< 0.0001			< 0.0001			< 0.0001
State Hospital	28.2	39.2		10.6	16.3		6.2	11.7	
O ther Inpatient	71.8	60.8		89.4	83.7		93.8	88.3	
Length of Stay (Days)			< 0.0001			< 0.0001			< 0.0001
1–7	30.6	24.9		52.8	49.9		63.1	64.6	
8–30	39.6	43.5		33.6	40.3		25.8	30.9	
31 +	29.9	31.6		13.6	9.9		11.1	4.6	

Among bipolar disorder patients, those with a timely outpatient admission (*N* = 3,995) were more likely to be younger and white than those not receiving such outpatient care (*N* = 41,418). They also had fewer diagnoses and were more likely to have a co-occurring substance use disorder. Finally, they more often had been inpatients at a state hospital and had an intermediate length of stay while there.

Turning to the group with a depressive disorder, those patients with an outpatient admission within 30 days (*N* = 6,732) were more likely than those without such an episode (*N* = 68,263) to be younger, female, white or of other/unknown race, of Hispanic ethnicity, and to have a co-occurring substance use diagnosis. Like the two other patient groups, they had fewer mental health diagnoses and were more likely to have been patients at a state hospital than another inpatient facility. Finally, they were more likely to have had a shorter length of stay while hospitalized.

### Patient Subgroups and Readmission Risk

**Fig. 1:**
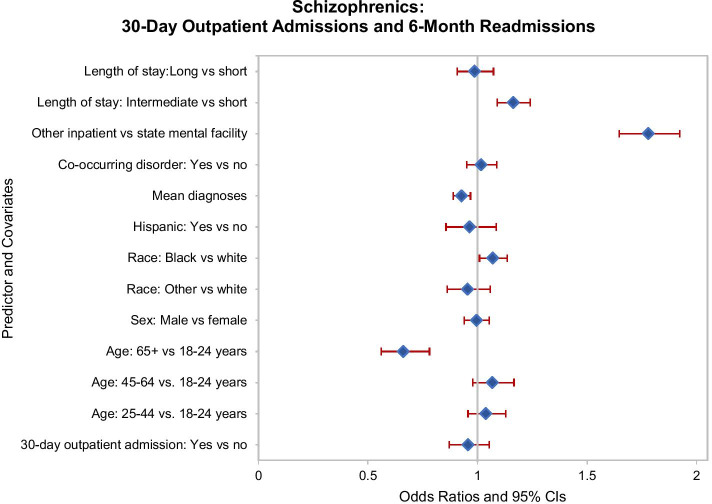
Schizophrenic disorder patients: Thirty-day outpatient admissions and readmissions during days 31–180 post-discharge. Odds ratios and 95% confidence intervals

Over days 31–180 following discharge, 13.3 percent of patients with a schizophrenic disorder were readmitted at least once. During the first 180 days, 15.7 percent of these patients had one or more outpatient treatment admissions, and within 30 days, 9.6 percent received the recommended outpatient treatment admission. In the multivariable logistic regression model, the subgroup with a 30-day outpatient admission was found to be no less likely to be readmitted over the next 31–180 days (adjusted odds ratio [AOR] 0.96, 95% CI 0.87–1.05; Fig. 1). Two subgroups were found to experience lower odds of readmission. Relative to patients with schizophrenia in the youngest age group, those in the oldest age group were less likely to be readmitted (AOR 0.66, 95% CI 0.56–0.78). Additionally, those patients with more mental health diagnoses were less likely to be readmitted (AOR 0.93, 95% CI 0.90–0.97). Several subgroups experienced a greater risk of readmission. Compared to White schizophrenic patients, Black schizophrenic patients were more likely to be readmitted (AOR 1.07, 95% CI 1.01–1.14). Relative to schizophrenic patients who were hospitalized at a state hospital, those hospitalized at other inpatient facilities were strongly more likely to be readmitted (AOR 1.78, 95% CI 1.65–1.93). Finally, relative to schizophrenic patients with the shortest length of stay, those with an intermediate length of stay were likelier to be readmitted (AOR 1.16, 95% CI 1.09–1.24).

**Fig. 2:**
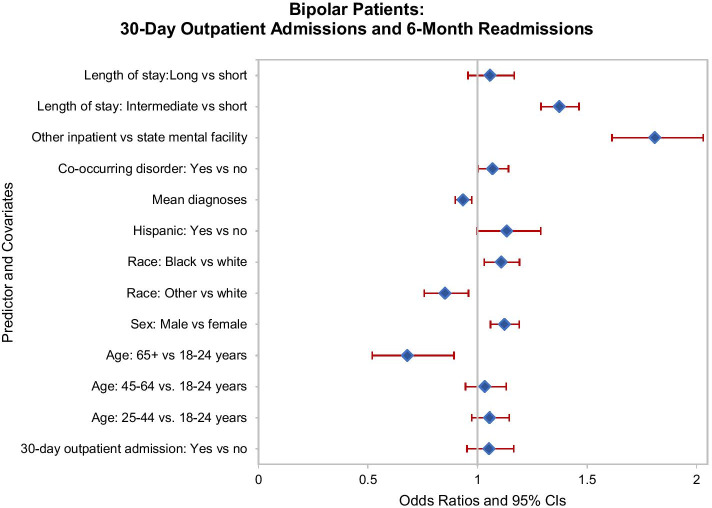
Bipolar disorder patients: Thirty-day outpatient admissions and readmissions during days 31–180 post-discharge. Odds ratios and 95% confidence intervals

Among the cohort with bipolar disorder, 11.4 percent of patients were readmitted at least once during days 31–180 following discharge. Of the total sample, 14.5 percent had one or more outpatient admissions in the 180 days following discharge, and 8.8 percent of patients were admitted as outpatients within 30 days of discharge. The subgroup with a 30-day outpatient episode, however, was no less likely to be readmitted during the subsequent 31–180 days than were those not receiving outpatient care in that time frame (AOR 1.05, 95% CI 0.95–1.17; Fig. 2). Several subgroups experienced lower odds of readmission. Relative to the youngest patients with bipolar disorder, those in the oldest group were less likely to be readmitted (AOR 0.68, 95% CI 0.52–0.89). Additionally, those with other/unknown race relative to being white were less likely to be readmitted (AOR 0.85, 95% CI 0.76–0.96), as were those with more mental health diagnoses (AOR 0.94, 95% CI 0.90–0.97). Several other subgroups faced higher odds of readmission, including Blacks (relative to whites; AOR 1.11, 95% CI 1.103–1.19), males (relative to females; AOR 1.12, 95% CI 1.06–1.19), and those with a co-occurring substance use disorder (AOR 1.07, 95% CI 1.00–1.14). As with patients with a schizophrenic disorder, two groups of patients with bipolar disorder had greater risk of readmission: patients who stayed at another type of inpatient hospital, compared to a state hospital (AOR 1.81, 95% CI 1.61–2.03), and patients with an intermediate length of stay (AOR 1.37, 95% CI 1.29–1.46).

**Fig. 3:**
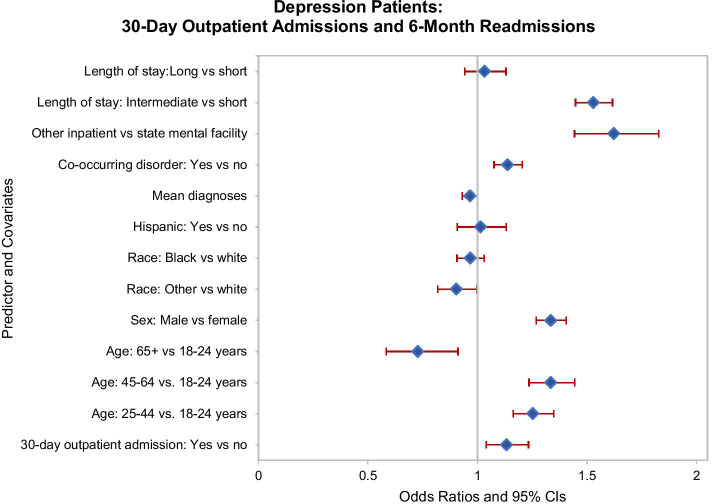
Depressive disorder patients: Thirty-day outpatient admissions and readmissions during days 31–180 post-discharge. Odds ratios and 95% confidence intervals

Finally, we turn to the cohort that had been hospitalized with depression, 9.0 percent of whom were readmitted at least once during the study period. Among this cohort, 14.0 percent had one or more outpatient admissions within 6 months of discharge and 9.0 percent had an outpatient admission within 30 days. This subgroup was slightly but significantly *more likely* to be readmitted over the 31–180 days post-discharge than were depressed patients without a 30-day outpatient admission (AOR 1.13, 95% CI 1.04–1.23; Fig. 3). Two subgroups among this cohort were less likely to be readmitted: those in the oldest age group (relative to the youngest patients; AOR 0.73, 95% CI 0.58–0.91) and those of other/unknown race (relative to whites; AOR 0.90, 95% CI 0.82–0.99). In contrast, several other subgroups of depressive patients were more likely to be readmitted, including those in the middle two age groups (relative to the youngest group; 25–44 years: AOR 1.25, 95% CI 1.16–1.35; 45–64 years: AOR 1.34, 95% CI 1.24–1.44), those who were male (AOR 1.34, 95% CI 1.27–1.41), and those with a diagnosis of co-occurring substance use disorder (AOR 1.14, 95% CI 1.08–1.20). As with patients with schizophrenic and bipolar disorders, non-state hospital patients with a depressive disorder were more likely to be readmitted (AOR 1.62, 95% CI 1.44–1.83). Last, depressed patients with an intermediate length of stay were more likely to be readmitted (AOR 1.53, 95% CI 1.45–1.62).

## Discussion

This is the first study to examine the effect of timely outpatient treatment on risk of readmission in serious mental illness patients who had been hospitalized in state-funded or -operated facilities. It is also among the few studies to examine readmission risk by whether timely outpatient care was received more generally. In previous studies of this latter question, there was some evidence that 30-day outpatient visits prevented readmissions. In contrast to those findings, after the analyses controlled for several potentially confounding factors, we found no evidence that receiving timely outpatient care reduced readmissions for patients suffering from schizophrenia or bipolar disorders and found that it was associated with increased readmissions for depressive patients. Finally, few patients in the sample received follow-up care within the recommended time frame despite the fact that it has been a quality indicator for many years. Together, these findings suggest the need for improvements in patient care processes at state-funded or state-administered facilities and programs.

An important and novel finding of the current study is that patients who stayed at a state hospital (14% of the sample) were much more likely to receive a follow-up outpatient admission within 30 days, and were much less likely to be readmitted, than those who stayed at another type of inpatient facility (86% of the sample). The reduction in readmissions for state hospital inpatients may be mediated by length of stay. For state hospital inpatients, the median length of stay was 34 days, whereas for patients hospitalized at other inpatient facilities, the median length of stay was only 7 days. Several prior studies have found that longer lengths of stay, or related measures such as adequate symptom resolution before discharge, are associated with a lower risk of readmission [e.g., 5, 6, 13, 14]. Patients in other inpatient facilities may have been more likely to be readmitted because they received inadequate treatment while hospitalized.

The above findings regarding state hospitals and longer stay lengths may help explain the lack of reductions in readmissions by outpatient visits in this sample. Among all three patient cohorts, those who had a follow-up outpatient visit within 30 days were also more likely to have been hospitalized at a state hospital. The lack of a benefit on readmissions may relate more directly to hospitalization at other inpatient facilities that receive state funds.

This study’s finding that depressive patients receiving the recommended follow-up care were more likely to be readmitted builds on prior studies that found no evidence that the recommended outpatient treatment reduced readmissions in this patient group [4, 7]. The literature on stay lengths and readmissions may also help explain why patients with depressive disorders and a 30-day outpatient admission had a greater readmission risk in the present study. Of the three patient cohorts, patients with depressive disorders had the highest percentage of short stay lengths. This did not appear to be because they were less sick; depressive patients were comparable to schizophrenic and bipolar patients in their number of diagnoses and prevalence of co-occurring disorders. Despite the large size of the study sample, depressed patients receiving the recommended outpatient treatment may have received inadequate care while hospitalized. However, both groups of depressive patients—those receiving follow-up care within 30 days and those not receiving it—had a high proportion of short stay lengths. Among depressive patients, having one or more physical comorbidities, which was unmeasured in MH-TEDS, is associated with increased readmissions [15]. More generally, factors that often affect depressive patients, including living alone and not having a regular primary care physician, are associated with increased all-cause 30-day readmissions [16, 17]. These factors may help explain why the current study found increased readmissions in patients with depressive disorders, and why prior studies have found that timely follow-up outpatient visits as a minimum do not appear to reduce readmissions among depression inpatients [4, 7].

We also found extremely low rates of outpatient treatment within 30 days of discharge within this sample of patients from state-funded and -operated facilities, with rates of 9–10 percent across the three clinical cohorts. This stands in contrast with rates of 30-day outpatient visits in studies finding a significant inverse association between timely outpatient treatment and readmission risk. For example, in Marcus et al.’s study, 64–73 percent of schizophrenic and bipolar patients received follow-up outpatient care within 30 days of discharge [9]. Okumura et al. found an even higher rate of follow-up outpatient treatment: 85 percent of the schizophrenic and bipolar patients they studied received such care [8]. It is possible that in environments in which many patients receive such follow-up treatment, the failure to administer such care may adversely affect readmission risk. We note that in contrast to our rates of timely follow-up outpatient treatment, readmission rates in the current study resembled those of Marcus et al. and Okumura et al.

This study is subject to several limitations in addition to the potentially confounding factors noted earlier. First, because the study used MH-TEDS data, it may not always have been the case that outpatient treatment occurred if an outpatient admission occurred; e.g., some states may have used the date for which treatment was scheduled (and not necessarily received) as their admission date. This quirk of the data set may have rendered the study conservative in its ability to find a benefit of timely outpatient treatment. However, we note that most states contributing data to MH-TEDS capture the actual start date of treatment in their admissions. Second, the data set included little information about how sick patients really were, other than their length of stay, number of mental health diagnoses, and the presence of a co-occurring disorder. Among our large sample, patients receiving timely outpatient care were less sick in terms of their mean number of mental health diagnoses but sicker in terms of their prevalence of co-occurring substance use. Third, we could not assess other outcomes of interest regarding the potential benefit of outpatient follow-up treatment, such as suicide risk. Fourth, the data were not nationally representative because they came from 13 states, a majority of which were Southern or Mid-Atlantic. Finally, the findings may not be generalizable beyond state-funded or -operated treatment. However, we note that in states such as Pennsylvania that contributed much data to the sample, a majority of mental health programs are administered or funded at the state (or more directly, the local) level [e.g., 18].

It is important to avoid interpreting these findings as indicating that timely follow-up outpatient care lacks a benefit in this group receiving state-facilitated care. It is possible that it improves other outcomes of interest, such as reducing depressive, manic, or psychotic symptoms; reducing suicide risk; or improving quality of life. Relatively few studies have examined these other potential benefits. However, Fontanella et al. [19] recently found that receiving timely outpatient care post-discharge was associated with a reduced risk of suicide among youth psychiatric inpatients.

In conclusion, follow-up outpatient care for psychiatric inpatients may need to be reevaluated because it did not appear to reduce readmissions, and for patients with depressive disorders it was associated with increased readmissions. Moreover, readmissions were more frequent than timely outpatient admissions, and lengths of stay were extremely short at non-state hospitals, which comprised the majority of the sample. These findings suggest that care processes in many state-funded and -administered hospitals and outpatient treatment facilities need to be improved.

## Data Availability

We used the restricted data analytic files of the MH-TEDS data sets for years 2014–2019. Some variables in this data set are not publicly available due to ethical restrictions. The public use data files are available through 2017 from the Substance Abuse & Mental Health Services Administration’s (SAMHSA’s) Drug and Alcohol Services Information System (DASIS) website: https://wwwdasis.samhsa.gov/dasis2/teds.htm.

## Code Availability

SAS 9.4 code is available from the first author.
